# Caring for Patients and Technological Competency in the Use of the Electronic Nursing Record System: A Qualitative Study

**DOI:** 10.3390/healthcare14010026

**Published:** 2025-12-22

**Authors:** Cvetka Krel, Dominika Vrbnjak

**Affiliations:** Faculty of Health Sciences, University of Maribor, 2000 Maribor, Slovenia; dominika.vrbnjak@um.si

**Keywords:** electronic nursing records, caring behaviour, technological competency, grounded theory, nursing documentation, qualitative research

## Abstract

**Background:** The purpose of using an electronic nursing record system (ENRS) is to support comprehensive and accurate nursing documentation and ensure safe and high-quality patient care. This qualitative study aimed to gain an in-depth understanding of nurses’ perceptions and the interrelationships between the effectiveness of ENRS, caring behaviour, and technological competency in nursing practice. **Methods:** Corbin’s and Strauss’s grounded theory approach was employed. Data were collected through semi-structured interviews with eleven nurses from four Slovenian hospitals, recruited using theoretical sampling between July 2021 and July 2023. Open, axial, and selective coding were conducted using MAXQDA 2020. **Results:** The core category “Losing caring in technology-focused documentation” was developed, including seven categories and seventeen subcategories. The paradigmatic model identified causal conditions (inadequate ENRS effectiveness), contextual conditions (poor ENRS integration into hospital environments), and intervening conditions (patient care) that contributed to insufficient documentation of individualised and holistic care. Two action–interaction strategies were identified: developing technological competency and integrating caring into documentation practices. Consequences manifested at individual, organisational, and national levels. **Conclusions:** Nurses’ caring behaviour, effective ENRS, and technologically competent use of the ENRS are essential for documenting and ensuring individualised and holistic patient care.

## 1. Introduction

The use of Electronic Nursing Record Systems (ENRS) plays a vital role in enhancing the quality of nursing care documentation, patient safety, and user satisfaction. Previous studies have confirmed that the effectiveness of ENRS is closely linked to system quality, usability, and user satisfaction, as well as the interrelationships among these components [1,2]. Recent research has identified specific challenges that compromise the effectiveness of ENRS, including data redundancy, poor workflow navigation, and cumbersome data entry processes [3]. Furthermore, interacting with ENRS reduces direct patient–nurse interaction time [4]. Focusing solely on technology-provided information can lead to overlooking caring for patients, which is fundamental in nursing [5,6]. Developing technological knowledge, skills, and evaluative competence while maintaining caring as central to nursing practice is essential [7]. Locsin’s middle-range Theory of Technological Competency as Caring in Nursing (TCCN) conceptualises technology as a means of expressing caring rather than replacing it. Through technological knowing, nurses can better understand patients as whole persons—unique, holistic, and complete—by integrating technology with empirical, personal, aesthetic, ethical, and technological ways of knowing [8,9]. In this study, the TCCN theory served as the theoretical framework for exploring how nurses in clinical practice achieve technologically competent use of the ENRS while simultaneously integrating caring for the patient. Caring behaviour, which involves holistically attending to patients’ needs, forms the foundation of nursing practice [10,11], and can guide the integration of caring language into electronic records, ensuring that documentation reflects both clinical data and the nurse–patient caring relationship [12].

While TCCN theory has been applied in various nursing contexts [13,14,15,16,17], this study applies TCCN specifically to ENRS use, which has not been sufficiently explored [13]. In previous qualitative EHR (electronic health records)/ENRS-focused studies, research has primarily examined nurses’ perceptions and experiences of nursing and electronic documentation, with an emphasis on documentation quality, patient safety, workload, organisational influences, and system-related challenges associated with the use of ENRS and EHR systems [18,19,20,21]. However, these studies have not explicitly examined how nurses integrate technological competency with caring behaviour within ENRS use, nor have they applied a caring-based theoretical framework such as TCCN to this process. This study addresses this gap by exploring the dynamic relationship between ENRS effectiveness, technological competency, and caring behaviour. Furthermore, the Slovenian healthcare context is characterised by the nationwide introduction of ENRS with partial implementation and limited integration with the EHR. This results in fragmented and heterogeneous documentation requirements, representing a distinct practice environment compared to previously studied settings. This warrants an investigation into how nurses navigate the integration of technological competency and caring when using ENRS [22]. Understanding the processes and conditions under which nurses maintain caring behaviour while effectively using ENRS is essential for improving both documentation quality and patient care outcomes.

This study, therefore, aimed to answer two questions:

(1) How do nurses in a hospital setting perceive the effectiveness of the ENRS, technological competency in its use, and caring behaviour?
(2) How is technological competency in nursing related to caring behaviour and the effectiveness of the ENRS?

## 2. Materials and Methods

### 2.1. Study Design

This qualitative study was part of a larger mixed-methods research project that investigated the effectiveness of the ENRS, the caring behaviours of nursing staff, and their technological competency, as well as examined the interrelationships among these factors within the context of ENRS use [23]. The qualitative component was conducted using a grounded theory based on Corbin and Strauss’s approach, which integrates both deductive and inductive reasoning [24], and enables the description of actual phenomena in the nursing care environment. The study is reported in accordance with COREQ [25].

Although grounded theory usually avoids a predetermined theoretical framework, Corbin and Strauss’s approach uses its inclusion when new or refined concepts are sought [26,27,28]. In this study, Locsin’s TCCN served as a sensitising rather than a prescriptive framework, guiding the development of the interview guide and initial conceptual focus while remaining open to emergent findings.

### 2.2. Study Setting and Sample

This study included four Slovenian hospitals using the same ENRS. Participants were nursing professionals holding secondary, higher, or postgraduate degrees who actively used ENRS in their professional practice. Inclusion criteria were: (a) secondary, undergraduate, or postgraduate nursing education; (b) employment at the hospital for more than three months with active ENRS use; and (c) capacity to contribute to understanding of the research topic. Exclusion criteria were: (a) individuals without the specified nursing qualifications; (b) employment of less than three months; and (c) non-use of ENRS in daily practice. No age restrictions were applied.

For the pilot study, a purposive sample of three nursing professionals was selected, consistent with sample sizes recommended for grounded theory pilot studies. For the main study, theoretical sampling was applied [24,26]. Participants were identified and invited by department head nurses based on their expertise and skills. Sampling was guided by ongoing data analysis, with sample size determined during data collection. Data collection concluded when theoretical saturation was reached, indicated by the absence of new information and a precise definition of emerging concepts [24,29,30]. All participants who met the inclusion criteria provided informed consent and remained in the study until completion, with no refusals or dropouts.

### 2.3. Data Collection

Data were collected through semi-structured interviews. A pilot study was conducted in July 2021 to test the interview technique and refine the sequence of questions. The main study interviews were conducted between September 2021 and June 2023, and the pilot interviews were included in the final analysis. The interview guide was developed based on: (a) literature review; (b) quantitative phase data analysis; and (c) insights from the three pilot interviews. Two nursing care experts, educated in caring theories, reviewed the guide, followed by a constructive discussion and revision. Interview questions were further refined during data collection based on emerging findings, consistent with the principles of grounded theory [24,26,31].

The interview questions focused on the demographic characteristics of nursing professionals and addressed all three key concepts under investigation: caring, technological competency, and the effectiveness of ENRS (see Appendix A).

Sampling, data collection, and data analysis were conducted simultaneously and interactively, incorporating constant comparative analysis [24,26]. Due to this approach, only 2–3 weekly interviews were conducted.

Interviews were conducted by the first author, a registered nurse and PhD candidate with clinical experience in the hospital, under the supervision of an associate professor with expertise in nursing and caring science. The interviewer had prior professional relationships with five participants through collaborative work at the hospital, while the remaining participants were not previously known to them. To minimise potential bias, a reflexive approach was adopted throughout data collection and analysis, including role clarification as a researcher rather than a colleague, reflexive memo-writing to document analytical decisions and assumptions, and critical discussion of emerging interpretations, and ongoing supervision by the co-supervisor. In addition, all transcripts were independently reviewed by the co-supervisor, an experienced researcher with expertise in qualitative methodology and caring theories, to enhance reflexivity and confirmability.

Participants were initially approached through department head nurses and subsequently contacted by the researcher via email or face-to-face to arrange interviews. Interviews were scheduled in advance and conducted one-to-one in private settings free from distractions. The purpose and procedure of the study were explained to the participants, including a clarification that they could withdraw from the interview at any point. Only the interviewer and participant were present during the interviews. The interviews were audio-recorded with the participants’ consent. In total, 420 min of interviews were recorded. The longest interview lasted 50 min, and the shortest 27 min. All recordings were transcribed verbatim. Each transcript was assigned a unique code, and all information that could reveal the identity of the hospital, the interviewee, or any other individuals mentioned during the interviews was removed.

### 2.4. Data Analysis

Data analysis followed Corbin and Strauss’s grounded theory approach, involving open, axial, and selective coding [24,26].

Open coding involved the decomposition, examination, comparison, conceptualisation, and categorisation of data, resulting in a comprehensive list of codes that were subsequently grouped into categories. Axial coding followed, during which the data from open coding were reassembled, and connections between codes and categories were identified. These relationships were established using the analytical strategy known as the paradigm model. The codes and/or categories were linked to the interview content to identify causes, patterns of interaction, and consequences. Selective coding integrated and synthesised key categories into a central overarching category.

Field notes captured observations regarding the interview process. Memos documented analytical reflections throughout coding—initially for individual codes, subsequently for subcategory and category development, and finally for core category formulation. During analysis, questions addressing sensitivity principles (e.g., what do these data signify), theoretical questions (e.g., are there connections between concepts), and practical questions (e.g., where are conceptual gaps) guided the process [26].

Data were coded by the first author. To enhance confirmability, the co-supervisor (PhD nurse researcher with experience in qualitative methodology) independently reviewed all transcripts, and coding consistency was verified through discussion. Discrepancies were resolved through consensus.

The data analysis process was facilitated using MAXQDA 2020 [32].

### 2.5. Trustworthiness

The trustworthiness of the research process was ensured by applying the four key criteria proposed by Lincoln and Guba: credibility, dependability, transferability, and confirmability [33]. The study’s credibility was also strengthened through adherence to grounded theory methodological principles and the authors’ prior experience with qualitative research. Credibility was ensured by representing the studied phenomenon as experienced by participants through accurate transcripts without additions or omissions. Transcripts were returned to participants for verification. The co-supervisor’s involvement in the research process further enhanced credibility. Dependability was ensured through thorough and consistent research process documentation, including sampling, data collection, and analysis. Analytical memo-writing was used throughout the coding process to document emerging insights, reflect on relationships between codes, and support the development of subcategories, categories, and the core category. Memos facilitated constant comparison and enhanced theoretical sensitivity during axial and selective coding. Transferability was supported by a detailed presentation and description of the findings, as well as by conducting interviews with nursing professionals in four different hospitals, selected based on the timeline of the ENRS implementation, which provided diverse contextual perspectives. Confirmability was achieved by meeting the criteria of credibility, dependability, and transferability, and by involving an additional researcher in data analysis and interpretation. Pilot interviews were included in the final dataset as they met the inclusion criteria, generated conceptually relevant data, and contributed to theory development. Their inclusion is consistent with grounded theory principles, which value all data that meaningfully inform the analytic process [24]. Theoretical saturation was considered achieved when ongoing data collection and analysis yielded no new properties, dimensions, or relationships between categories, and when the categories were conceptually well-developed and analytically robust.

### 2.6. Ethical Considerations

Ethical approval for the study was granted by the National Medical Ethics Committee of the Republic of Slovenia (approval number: 0120-335/2020/3, dated 19 August 2020). Written institutional consent was obtained from all participating hospitals. To ensure confidentiality and comply with data protection regulations, the identities of hospitals remain undisclosed. Participants were informed about the study’s aims and methodology prior to their participation. Written informed consent was obtained, emphasising the strict confidentiality of personal data and the right to withdraw at any stage. Confidentiality and anonymity were ensured by removing all identifying information before data processing.

## 3. Results

Eleven participants voluntarily participated in the study. The sample included three men and eight women. Seven participants were employed at hospitals where the ENRS had been used for less than 12 months and were still in the testing phase. The remaining four participants were employed at a hospital where the ENRS had been in use for more than 24 months and had been fully implemented into the hospital’s nursing documentation environment. Participants’ ages ranged from 24 to 58 years, with a mean age of 37. Their length of employment ranged from 6 months to 38 years, with an average of 13.7 years of work experience.

**Figure 1:**
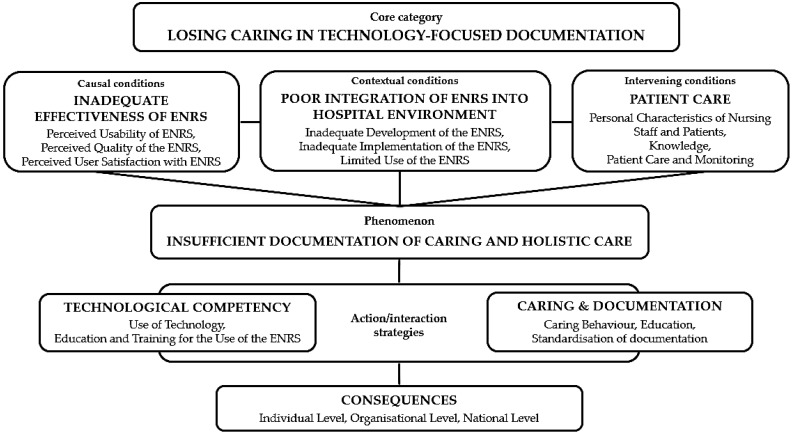
Paradigm model of losing caring in technology-focused documentation: categories and their relationships. Legend: ENRS—the electronic nursing record system.

Based on the analysis of interviews, field notes, and analytical memos, the core category “Losing caring in technology-focused documentation” was developed. Seven categories and seventeen subcategories were identified. A summary of categories, subcategories, and illustrative quotations is presented in Table 1. The paradigm model of losing caring in technology-focused documentation, including its categories and their relationships, is presented in Figure 1.

These categories and subcategories revealed complex relationships operating at individual, organisational, and national levels. The inadequate effectiveness of the Electronic Nursing Record System (ENRS) in terms of usability, quality, and user satisfaction, combined with poor integration of the ENRS into the hospital environment (due to inadequate development, implementation, and limited use), created conditions that led to insufficient documentation of caring and holistic care. These conditions were further influenced by factors related to patient care, including personal characteristics of nursing staff and patients, knowledge gaps, and staffing challenges. Two key strategies were developed to address this phenomenon: developing technological competency and integrating caring for the patient into documentation practices. The analysis revealed that when technology became the primary focus, caring was lost in the documentation process. This core category of “Losing Caring in Technology-Focused Documentation” connected all categories and explained how the interplay between system effectiveness, hospital integration, and staff factors resulted in fragmented documentation that focused primarily on physiological needs while omitting psychological, social, and spiritual dimensions of care. The consequences of this process manifested at multiple levels, affecting individual patients and nurses, organisational outcomes, and the broader healthcare system.

### 3.1. Inadequate Effectiveness of ENRS

An ENRS was considered effective when perceived as usable and of high quality, and when staff were satisfied with its use. Three subcategories were identified: usability, effectiveness, and satisfaction with the use of ENRS. The characteristics of the ENRS were assessed differently by the interviewees, with opinions varying between hospitals, as the system is not equally developed across all institutions.

#### 3.1.1. Perceived Usability of ENRS

A common issue across all hospitals was that the ENRS was not used for electronically documenting nursing shift handovers, patient transfers, and discharges, as not all hospital departments had access to the ENRS. This affects the continuity of patient care documentation, resulting in parts of the documentation being either missing or available only in paper form. The ENRS did not provide access to newly ordered tests and their results, as it was not integrated with the EHR (the electronic health record).

*“We do not use ENRS for documenting nursing shift handovers and patient transfers.”*

(B4)

*“… the ENRS currently does not provide access to newly ordered diagnostic tests or their results, as it has not yet been integrated with the EHR.”*

(A3)

The ability to select nursing diagnoses, goals, and interventions was identified as a positive feature of the ENRS. However, it was also found in all participating hospitals that specific nursing diagnoses required for documenting the nursing process were missing.

*“The ENRS was developed primarily based on outdated paper-based documentation, which does not adequately reflect the nursing diagnoses currently required in clinical practice.”*

(B6)

#### 3.1.2. Perceived Quality of the ENRS

The ENRS was perceived as high quality when it demonstrated speed, ease of use, consistent functionality, and the ability to provide accurate information promptly.

*“… it is not slow and rarely becomes non-functional, so I do not encounter any difficulties in its use …”*

(A1)

*“… it seems to me that it sometimes operates a bit slowly; otherwise, it ensures transparency of the data …”*

(C8)

*“… the ENRS provides accurate information, each user logs in with their code, and it is evident who performed which tasks for the patient …”*

(A9)

#### 3.1.3. Perceived User Satisfaction with ENRS

User satisfaction with ENRS varied across hospitals and was influenced by implementation status and system functionality. Satisfaction was lower in hospitals where the system had been implemented for less than 12 months, and only a trial version of the ENRS was used, primarily due to the requirement to document all patient care data twice on paper and electronically. The ability to document at the patient’s bedside and to enter data immediately after the nursing intervention was identified as an advantage, as it reduced the likelihood of omitting documentation of completed interventions and increased nurses’ physical presence with the patient.

*“… it is unfortunate that only a trial version is in use, which requires all nursing documentation to be recorded both on paper and electronically…”*

(A1)

*“… I can document directly in the hospital room at the patient’s bedside, allowing me to simultaneously ask the patient questions, document and maintain physical presence with them…”*

(B5)

### 3.2. Poor Integration of the ENRS into the Hospital Environment

Three subcategories were identified: inadequate development of the ENRS, insufficient implementation of the ENRS, and limited use of the ENRS. Shortcomings in the development, implementation, and use of the ENRS are attributed to national, organisational, and individual levels.

#### 3.2.1. Inadequate Development of the ENRS

Inadequate development, as well as the subsequent implementation and utilisation of the ENRS within the hospital setting, stemmed from a lack of interest, insufficient staffing, and limited funding dedicated to the digitalisation of nursing care at the national, organisational, and individual levels. Financial resources for healthcare digitalisation were disproportionately directed towards medical treatment, resulting in insufficient investment in nursing activities and patient empowerment in self-care management.

*“… further progress requires additional funding and a shortage of nursing staff. Additionally, healthcare funding for digitalisation and service reimbursement focuses on hospital patient care and reducing waiting times rather than patient empowerment and preventive care…”*

(D11)

*“… prolonged testing phase without expansion to other departments has diminished enthusiasm and reduced the willingness to invest effort in further developing the ENRS…”*

(A10)

#### 3.2.2. Inadequate Implementation of the ENRS

The ENRS was either not integrated or only partially integrated with the electronic medical record system. Interviewees confirmed that inadequate implementation of the ENRS resulted in incomplete documentation of the entire patient care process, leading to reduced transparency and comprehensiveness of patient data. The need to enter or search for patient information in the ENRS and the EHR increased the time required to document patient care. Only in hospitals where the ENRS had been implemented for more than 24 months was the system fully deployed across all departments; in other hospitals, electronic data exchange concerning nursing care related to patient management between departments was not ensured.

*“… integration of the ENRS with the EHR is necessary to consolidate all relevant information, including nursing assessments, test results, and patient categorisation, in one place. This would decrease documentation time and enhance the accessibility and comprehensiveness of patient information.”*

(B5)

#### 3.2.3. Limited Use of the ENRS

The included hospitals exhibited varying levels of ENRS development and implementation, influenced by national and hospital factors that affected its usage within the hospital environment. Consequently, nursing care was documented both on paper and electronically (at hospitals A, C, and D), resulting in duplicated work. Due to time constraints, not all collected data was entered into the ENRS. Documented patient data during transfers was not accessible to other departments and hospitals, impacting the continuity of patient care and employee satisfaction with the ENRS. Nursing staff thus lacked access to information about the patient’s care, previous hospitalisations in other hospitals, and healthcare institutions. Paper-based nursing documentation had to be retrieved from archives among extensive patient records, which hindered time effectiveness. Additional challenges with paper documentation included illegible data and the risk of data loss.

*“… because not all departments and hospitals have the ENRS, problems arise during patient readmissions. This process is time-consuming, as we search through files for information, and sometimes handwritten notes are difficult to read, or documents may be lost…”*

(A9)

The issue concerned not only the implementation of the ENRS within Slovenian healthcare institutions but also the standardisation of nursing care documentation. Interviewees reported challenges in obtaining comprehensive patient information during transfers from other institutions, noting that the available data were often incomplete and predominantly focused on patients’ physiological needs.

*“… when receiving patients from other institutions, different forms are often used, resulting in frequent gaps in information. Documentation during transfers primarily focuses on physiological needs, limiting the visibility of the patient’s comprehensive needs…”*

(A2)

### 3.3. Patient Care

Three subcategories were developed: personal characteristics of nursing staff and patients, knowledge and patient care and monitoring of care. This category was identified as an indirect condition that facilitates or impedes action–interaction strategies.

#### 3.3.1. Personal Characteristics of Nursing Staff and Patients

Nurses’ personal characteristics were also linked to their perceptions of the development, implementation, and use of the ENRS. Limited awareness among nursing staff regarding the importance of nursing documentation led to incomplete recording of all phases of the nursing process, resulting in insufficient documentation of overall patient care. The introduction of the ENRS and innovations in general often provoked resistance and rejection among employees. Interviewees also noted a lack of digital literacy among nursing staff, with some employees, particularly older ones, actively avoiding the use of the ENRS.

*“…principal cause lies in the attitudes of the nursing staff—more specifically, their general attitude toward modern technology and all innovations. When a new development is introduced, the reaction is: ‘Not now, maybe later…’ They fail to perceive the benefits…”*

(B6)

*“* *… even some registered nurses are not sufficiently computer literate, and some nurses avoid it as much as possible…”*

(C8)

The interviewees stated that, during data entry, they focused too much on using the ENRS rather than on the patient. Although they explained this by saying that the hospital required it, the analysis suggested that the reasons also lay with the nursing staff themselves, resulting in the inappropriate use of the ENRS. Consequently, the patient was not adequately involved in the care process.

*“… everything be documented in the ENRS, as extensively as possible, since we no longer have a paper format, and the actual implementation of the patient’s needs and services is forgotten. The focus is on the ENRS…”*

(B5)

Patient participation was also essential in inpatient care and should have been documented. If specific nursing interventions were not performed at the patient’s request due to refusal of care or changes in the patient’s health status, these were not documented. However, this did not mean that the patient was not provided with comprehensive care; rather, it represented an essential aspect of nursing documentation.

*“… we have a patient who is repositioned only onto their left side. And the right? The patient refuses. Where is this recorded in the documentation?”*

(B6)

#### 3.3.2. Knowledge

The interviewees believed that nursing staff require theoretical knowledge in the medical field, nursing care, nursing documentation, and the use of the ENRS to care for patients adequately. The interviewees expressed that they lacked sufficient knowledge and training in nursing documentation.

*“… It is necessary to have solid theoretical knowledge, not only general medical knowledge, but also competence in nursing documentation. Without a strong theoretical foundation, accurate and comprehensive documentation is not possible, and essential information may be omitted…”*

(B7)

*“… we still have insufficient knowledge and too few training opportunities in nursing documentation…* *”*

(A2)

#### 3.3.3. Patient Care and Monitoring

Nursing staff did not always follow standards and work instructions during patient care. Patient care deviated from professional guidelines and instead relied on what was easier or how colleagues performed tasks, which could be improved through regular monitoring (supervision) of patient care.

*“Staff do not always adhere to the established standards; instead, they often choose the easiest way to complete their tasks, similar to other employees. As a result, it is essential to verify patient care…”*

(B6)

Recruitment and employment of personnel primarily depended on hiring policies at both the organisational and national levels. While the ENRS enabled statistical analyses of completed work, the entire patient care process was not fully documented, making it difficult to provide accurate data for staffing norms. This was particularly unfeasible in hospitals where ENRS was used on trial. Staffing data were based on an outdated categorisation that had not been revised to reflect changes in patient care provision.

*“… Staffing allocation is based on categorization … according to the Slovenian categorization, it was supposed to be factored in, but the last calculation was done in 1990 when patient care was different than it is now … If we were to consider all this and recalculate according to current needs, we would see a shortage of two-thirds of the staff …”*

(B6)

Interviewees reported experiencing difficulties in using the ENRS due to staff shortages, which could adversely affect patient care and documentation. They noted that limited time often prevented meaningful conversations with patients, which could lead to an incomplete understanding of patient needs and affect the provision and documentation of individualised and holistic care.

*“… There is so much to enter into the computer that time becomes a problem … you focus on giving medications on time, and then the patient wonders why you cannot talk to them …”*

(C8)

Staff shortages were also perceived as a barrier to further development of the ENRS. Participants noted that employees involved in system development were simultaneously engaged in regular work duties, resulting in insufficient time to contribute effectively.

*“… I think there should be one person assigned exclusively to EHRS development, with sufficient time dedicated to it …”*

(C8)

### 3.4. Insufficient Documentation of Caring and Holistic Care

The ENRS did not support documenting individualised and holistic patient care, as it primarily focused on recording the patient’s physiological needs. Interviewees believed that much of the nursing work performed for patients remained undocumented. Deficiencies in documenting patient care also extended to the lack of recording information from nursing staff to patients’ relatives, physicians, and other healthcare team members involved in patient care. Some data were entered into the ENRS, while others were recorded in the EHR, resulting in fragmented, disconnected documentation that did not reflect a holistic patient care process.

*“… We document data mainly concerning physiological needs, such as bed baths, changing clothes, feeding the patient, wound dressings, … social and psychological aspects only partially.”*

(A3)

*“… It is not visible what physiotherapists and other hospital staff have done with the patient… now some information is written in Medis, some in our ENRS, and it is unclear, fragmented, disconnected, and does not represent the care process as a whole…”*

(A10)

Across all hospitals, interviewees reported that patient care documentation within the ENRS originated from a previous, outdated paper-based format, which failed to record all the patient’s life activities and, consequently, did not fully reflect the patient’s needs.

*“… in the electronic documentation itself, including the focus in the nursing history and the selection of interventions, we essentially follow the six activities according to Virginia Henderson …”*

(B6)

### 3.5. Technological Competency

Technological competency included two subcategories: use of technology (ENRS) and education and training.

#### 3.5.1. Use of Technology

The interviewees defined technologically competent use of the ENRS as the ability to retrieve patient information, interpret it appropriately, and apply it to provide holistic care. When documenting care, the ENRS included the patient’s needs, health status, and preferences. According to the interviewees, the technologically competent use of the ENRS enabled them to document the nursing process effectively.

*“… technologically competent use of the ENRS means that we incorporate the nursing process: identifying needs, creating a plan in the chart, adjusting interventions according to the patient’s health status, and finally evaluating the goals and the nursing care provided …”*

(B5)

In using technology, it was also necessary to consider the Code of Ethics. The interviewees emphasised that nursing staff were committed to preserving life and that technology should not take precedence over patient care, which related to the critical use of the ENRS.

*“… it is necessary to follow the Code of Ethics for Nurses and Nurse Assistants of Slovenia. When using the ENRS (*e.g.*, if a patient is in severe pain or their health status deteriorates), we should not focus on the tablet and the documentation process, but rather attend to the patient first …”*

(A9)

The interviewees also highlighted the importance of data. It is the responsibility of the hospital and the information system owner to establish and maintain an information system that safeguards patient data.

*“… whenever we enter a patient’s room, we log in with our username and password, which are intended for use by one individual only. After completing the assessment, we always log out … data protection must also be ensured by the information system itself …”*

(A9)

#### 3.5.2. Education and Training for the Use of the ENRS

In all hospitals, staff received education and training for using the ENRS, both from the owner of the information system and the person responsible for the ENRS within the hospital. The training influenced the use of the ENRS and its further development.

*“… at the beginning, there was considerable resistance to using the ENRS; it was something new, and people were naturally apprehensive about how it would work. However, we received training from the owner of the ENRS, and now we use it routinely and are also developing and adding new features …”*

(B5)

*“… we also receive support from the nurse educator, who trains us in the use of the ENRS …”*

(B7)

Staff can also acquire knowledge in digitalisation through training provided by the Nursing Informatics Section, which promotes the development of the ENRS in Slovenia.

*“… the Nursing Informatics Section also organises conferences where electronic documentation of nursing care is presented …” *

(A10)

### 3.6. Caring and Documentation

Caring and documentation included three subcategories: caring behaviour, education, and standardisation of documentation, using NANDA diagnoses (The North American Nursing Diagnosis Association), NIC (The Nursing Interventions Classification (NIC) and NOC (Nursing Outcomes Classification). These subcategories represented action–interaction strategies for integrating caring into documentation practices.

#### 3.6.1. Caring Behaviour

The interviewees recognised care as defined by caring theory but also expressed the view that not all nurses were caring. Caring could be an innate quality, influenced by early family socialisation, or acquired through professional development. The interviewees defined caring for patients as encompassing physiological, psychological, sociological, and spiritual needs. They ensured that the patient’s needs were met by focusing on them, considering their feelings, and maintaining a respectful and empathetic relationship.

*“In my opinion, not all nurses are caring … I believe caring is either innate or dependent on upbringing. It could also be acquired later …”*

(A9)

*“… caring behaviour for me means caring for the patient according to their physiological, psychological, social, and spiritual needs …”*

(A1)

*“… we consider the patient as a person, not just as an object; we maintain a respectful attitude towards the patient, support their hope and wishes, and take into account their inner feelings …”*

(A3)

The interviewees also believed that caring for the patient and ensuring basic life activities include providing health literacy and empowerment to enhance the patient’s self-care.

*“… caring for the patient means ensuring the patient’s basic life needs, providing health education, improving health literacy, and empowering the patient regarding their illness and health status, to increase self-care according to their abilities and condition assessment …”*

(D11)

The interviewees also recognised the need to involve nursing staff with caring attributes in developing the ENRS. Only in this way would the ENRS enable the documentation of holistic patient care rather than focusing primarily on documenting physiological needs.

*“… nurses involved in the development of the ENRS should possess caring attributes; otherwise, it will not be possible to document the reasons for patients’ distress and provide in-depth care. Instead, we will continue to document mainly the performed physiological needs, as has been the case so far …”*

(A9)

#### 3.6.2. Education

To provide caring for patients, nursing staff needed appropriate knowledge, which they acquired through education and training. In the hospital setting, staff gained knowledge through online courses and internal and external training sessions. According to the interviewees, patient care could also be enhanced through education and team meetings to establish a culture of caring within the hospital.

*“… caring behaviour could also be developed subsequently through education or team discussions, to promote a culture of caring on the wards …”*

(A9)

#### 3.6.3. Standardisation of Nursing Care Documentation

In nursing, it was necessary to introduce standardised language into the ENRS, which should be based on theoretical foundations and link theory with practice. The existing documentation was primarily derived from outdated paper-based forms, which differed from hospital to hospital. Standardised documentation and language in nursing could contribute to the harmonisation of records not only at the organisational level but also at the national level.

*“… we should all have the same standardised forms prepared. Each hospital develops them in its way, which leads to deficiencies in the forms and results in much missing data of patient care …”*

(A9)

*“… it is necessary to implement a standardised approach to nursing documentation, such as NOC, NIC, and NANDA, supported by an integrated information system that enables the selection of nursing diagnoses and prioritises those most relevant to the patient. This would provide an overview and allow for setting goals and interventions accordingly. At present, it is not possible to adequately evaluate my work, as appropriate goals are not even established …”*

(B6)

### 3.7. Consequences

Consequences were categorised at individual, organisational, and national levels, focusing on caring for the patient and documenting holistic care through technologically competent use of the ENRS.

#### 3.7.1. Individual Level

The consequences related to patients and their health. According to the interviewees, caring for patients and documentation of their care had a direct impact on the quality and safety of care, patient health, and treatment outcomes. Deficiencies in this area could lead to complications and deterioration in patient health. Inadequate caring and documentation when using the ENRS could also have consequences for the nurses providing care. The interviewees noted that caring behaviour in nursing practice reduced the occurrence of errors in medication administration and mistakes in information transmission.

*“… if we do not incorporate caring behaviour into patient care, this is reflected in the patient’s health status, treatment outcomes, and complications such as falls and infections …”*

(A10)

The interviewees reported that incorporating caring behaviours into patient care had a positive impact on patient satisfaction. Caring behaviour extended beyond addressing physical needs, encompassing an individualised and holistic approach that supported psychophysical wellbeing. Such an approach fostered patients’ sense of being acknowledged and respected, which could enhance their trust in healthcare providers, facilitate treatment acceptance, and contribute to improved treatment outcomes.

*“… I think that patients are more satisfied when we include caring behaviours alongside physical care, because they feel that we pay more attention to them and respect them as individuals… because we take their wishes into account, they feel recognised, we encourage their hope, so they are more likely to accept information and treatment …”*

(A3)

#### 3.7.2. Organisational Level

Deficiencies in caring and documentation could lead to patient and family dissatisfaction, complaints, lawsuits, and, consequently, financial costs for the hospital. The interviewees linked the effectiveness of ENRS and caring practices with providing holistic patient care and its documentation. In their opinion, the combination of caring patient care and effective ENRS could ensure continuity of care, improve the quality of work, increase patient safety, and create better working conditions for nursing staff.

*“… we should have a uniform, efficient ENRS and use it in a technologically competent way to ensure proper documentation, while also integrating caring for the patient. All of this can impact the quality and safety of patient care…”*

(A10)

The ENRS allowed for partial documentation of nursing activities, which limited the ability to produce comprehensive reports that demonstrate holistic care provision. As a result, statistical data were incomplete and could not be reliably used for workforce planning or changes in job categorisation.

*“… the work of nurses will only be evident and recognised when everything is documented …”*

(B6)

#### 3.7.3. National Level

Litigation could also damage the reputation of hospitals and weaken public confidence in healthcare professionals. The interviewees highlighted that if all hospitals had a uniform ENRS connected across institutions, it would be possible to monitor the continuity of patient care, ensure necessary information about patients, and reduce errors in care.

*“… if there are many complaints in a hospital, it is perceived nationwide as a poor institution, and people lose trust. A uniform ENRS connected across institutions would reduce errors in care…”*

(A10)

The analysis revealed deficiencies in ENRS and caring behaviour that could result in compromised quality and safety of patient care, inadequate legal protection of nursing activities, and the inability to generate statistical reports needed to determine staffing needs at the national level.

*“… if the ENRS was efficient and applied appropriately, comprehensive care could be delivered in any Slovenian healthcare facility, ensuring continuity, quality, and real-time documentation while safeguarding safety…”*

(D11)

## 4. Discussion

The findings suggest that technology-focused documentation systems may undermine holistic patient care when system effectiveness, organisational integration, and caring competencies are misaligned.

The effectiveness of the ENRS was evaluated in terms of its usability, quality, and user satisfaction, as well as the interrelationships among these components [2,34,35,36]. The quality of the ENRS in our study was primarily associated with system functionality, accessibility, and clarity of information. At the same time, shortcomings were reported regarding the sufficiency, relevance, and completeness of data [34,37]. These findings are consistent with prior qualitative research, which shows that nurses perceive high-quality nursing documentation as essential for effective clinical communication, continuity of care, and patient safety. However, they also report persistent gaps between documentation standards and routine practice, often related to workload pressures and limited organisational support [18]. Previous studies similarly highlighted strengths in ease of documentation and medication accuracy, which support patient safety, but noted gaps in communication and usability [35,38,39].

Similar benefits of ENRS use were identified in our study, as reported in previous research [40,41]. As one participant noted, bedside documentation enabled nurses to stay with the patient while simultaneously accessing and recording essential information. Using ENRS provided timely access to relevant data, such as planned interventions (wound care, pressure ulcer prevention, etc.), which could contribute to greater efficiency and continuity of care. However, time efficiency depended on contextual factors such as nursing experience, patient condition, and hospital setting characteristics [42]. Comparable findings indicate that improved access to clinical information and perceived gains in patient safety are often accompanied by increased documentation workload, technostress and greater demands for training and organisational preparedness [19,21].

The study also revealed insufficient use of ENRS, as it did not support shift handover documentation or the recording of patient information during intra- or inter-hospital transfers, which interviewees found to be its most disturbing limitation. In our study, satisfaction with the ENRS was defined by its use. Overall, the system was perceived positively, as improvements were reported in information quality, work performance and patient safety, while its implementation within the hospital was considered insufficient. This finding aligns with broader evidence indicating that limitations in interoperability, documentation completeness, and system integration continue to hinder the effective use of ENRS in clinical practice [20,21]. Within the Slovenian nursing environment, our findings indicate that organisational and workflow conditions play an important role in shaping everyday ENRS use, with these limitations being particularly pronounced due to partial ENRS implementation and the absence of integrated functionality to support handover and patient transfer documentation, which directly affects continuity of care. Electronic records significantly reduce documentation errors and improve the quality of patient care [39]. Easier access to information and more efficient documentation were reported as sources of satisfaction, while electronic records were confirmed to contribute to higher documentation quality and, consequently, better patient care [41,43].

In this study, technological competency was understood as acquiring patient information, interpreting it appropriately, and applying it to individualised and holistic care. It was emphasised that, in competent use of the ENRS, the patient remained the focus rather than documentation or technical problem-solving. Previous studies have shown that data entry time can hinder caring for patients and generate staff resistance [44]. Although nurses spend more time in patient rooms after EHR implementation, this does not necessarily improve caring for patients [40]. While ENRS use may increase efficiency, it may also distract from patient-centred care unless aligned with nursing principles [13].

Ethical considerations were regarded as essential, in accordance with the Slovenian Code of Ethics for Nurses. Training and education were crucial for the competent use of ENRS, influencing current practice and further system development. Education should provide empirical knowledge in both nursing and basic medicine. Proper infrastructure and training enabled nurses to acquire both technological skills and an ethical foundation [13]. Interviewees noted that additional knowledge could be gained through professional nursing informatics education in Slovenia; however, they deemed training in nursing documentation to be insufficient. Universities were considered essential in preparing students for the competent use of electronic records. Nurses’ personal characteristics, including their caring disposition, level of knowledge, and staffing norms, could significantly influence the development, implementation, and use of ENRS. Although an efficient ENRS is an essential prerequisite for accurate and comprehensive documentation, it does not guarantee the documentation of holistic patient care. This finding extends previous research by demonstrating that earlier studies focused primarily on EHR efficiency, rather than on the documentation of individual and holistic patient care. To enable the documentation of individual and holistic patient care, the ENRS use must be supported by nurses’ caring attitudes and technological competency.

Findings from the interviews suggested that caring behaviour entailed addressing the patient’s physiological, psychological, social, and spiritual needs, thereby enabling knowing the patient as a whole person. Caring for the patient represented the foundation of patient care and was aligned with Jean Watson’s Theory of Human Caring [45,46]. The use of technology, including viewing the patient as a whole person, has been emphasised in Locsin’s theory [8] and subsequent studies [13,44,47]. Our findings extend Locsin’s theory by showing that technological competency in ENRS supports knowing the patient as a whole only when it is actively integrated into caring behaviour during everyday documentation. In this way, ENRS use becomes part of the caring process rather than a separate technical task.

Our study and others studies described continuous collaboration among nurses, patients, and IT specialists as necessary for effective ENRS use and the delivery of caring for patients [13]. Our study emphasised that technological competency in ENRS was insufficient without caring behaviour, as otherwise patient needs may remain unrecognised and deficiently documented. Other studies have confirmed that ENRS use should support, rather than hinder, nursing workflows, consistent with Locsin’s principle that technology must enhance nurses’ ability to know the patient as a whole [13,22]. This interpretation is supported by qualitative evidence from connected health research. Chen et al. (2020) demonstrated that technology shapes care processes and professional roles by influencing how healthcare professionals interact with patients and clinical information, supporting accessibility, continuity and personalised care only when functioning as a well-integrated technological platform within a coordinated multistakeholder ecosystem [47]. These findings suggest that digital documentation tools such as ENRS support caring practice when nurses integrate technological competency with caring intentions as part of their everyday care processes.

This study has several limitations that should be considered when interpreting the findings. The small sample from four Slovenian hospitals limits the transferability of the results, and the national context may further restrict applicability to settings with different ENRS implementations. The findings also represent only the perspectives of nurses, which may not fully capture the experiences of other stakeholders involved in ENRS use. Moreover, the shorter implementation period in some hospitals constrains conclusions regarding long-term effects. Despite these limitations, the study provides valuable insights into the interplay between technological competency, caring behaviour, and ENRS effectiveness.

The findings of this study have important implications for nursing practice, education, system design, and healthcare policy. Implications for practice highlight the need for technologically competent use of the ENRS that consistently integrates caring behaviour into documentation processes. Nurses should be supported in using the ENRS not merely as a technical tool for recording tasks, but as a means of documenting individualised and holistic patient care that encompasses physiological, psychological, social, and spiritual needs. Bedside documentation, when used critically and ethically, may support continuity of care while maintaining the nurse–patient relationship as central to practice.

Implications for nursing education emphasise the importance of integrating technological competency with caring theory throughout undergraduate and postgraduate curricula. Nursing education should prepare students to critically and responsibly utilise electronic documentation systems while maintaining a caring foundation as the basis of nursing practice. Ongoing professional education and reflective team discussions are also essential to strengthen caring behaviour, documentation competencies, and ethical awareness in clinical settings.

Implications for system design indicate that ENRS development should actively involve nurses who possess both technological competency and caring attributes. The system should support the documentation of holistic care rather than focusing predominantly on physiological needs. Full integration of the ENRS with the EHR, alongside the implementation of standardised nursing language (e.g., NANDA, NIC, NOC), is essential to ensure comprehensive, comparable, and transparent documentation across institutions and levels of care.

Implications for healthcare policy point to the need for national standardisation of nursing documentation and uniform implementation of ENRS across healthcare institutions. Adequate funding and strategic support for nursing digitalisation are required, with particular attention to staffing norms, education, and patient empowerment. At the policy level, recognising nursing documentation as a key component of patient safety, quality of care, and legal accountability is crucial for strengthening continuity of care and public trust in healthcare systems.

Future studies should investigate how interdisciplinary collaboration among nurses, IT specialists, and hospital managers influences the adoption of ENRS, supports their effective integration into clinical workflows, and strengthens the system’s capacity to ensure holistic patient care documentation. Moreover, further studies are needed to explore how the use of ENRS impacts interprofessional communication and patient care, as well as the extent to which these systems enhance the quality, safety, and continuity of patient care.

## 5. Conclusions

The findings demonstrated that individualised and holistic patient care required nurses’ caring behaviours, an effective ENRS that enabled documentation of all patient needs within the nursing process, and technological competency for its critical and responsible use. Without these elements, documentation risked remaining incomplete, and patient needs could be insufficiently recognised and treated. These findings have important implications for nursing education, ENRS development, and healthcare policy in Slovenia and beyond.

## Figures and Tables

**Table 1:** Overview of the categories, subcategories, descriptions and illustrative quotations.

Category	Subcategory	Description	Exemplar Quotation
Inadequate effectiveness of the ENRS	Perceived usability of ENRS	Limited functionality, lack of EHR integration, and incomplete support for the nursing process resulted in fragmented documentation.	“We do not use ENRS for documenting nursing shift handovers and patient transfers.” (B4)
	Perceived quality of ENRS	ENRS quality was associated with system speed, reliability, and data clarity, with variability across hospitals.	“… it is not slow and rarely becomes non-functional …” (A1)
	User satisfaction with ENRS	Satisfaction depended on the implementation stage and the requirement for dual documentation.	“… it is unfortunate that only a trial version is in use …” (A1)
Poor integration of the ENRS into the hospital environment	Inadequate development of ENRS	Limited funding, staffing shortages, and national priorities hindered the development of ENRS.	“… further progress requires additional funding …” (D11)
	Inadequate implementation of ENRS	Partial implementation and lack of ENRS–EHR integration increased the documentation burden.	“… integration of the ENRS with the EHR is necessary …” (B5)
	Limited use of ENRS	Continued paper documentation and lack of interoperability disrupted continuity of care.	“… handwritten notes are difficult to read …” (A9)
Patient care	Personal characteristics of nursing staff and patients	Nurses’ attitudes, digital literacy, and resistance to change influenced the use of ENRS and patient involvement.	“… some nurses avoid it as much as possible …” (C8)
	Knowledge	Insufficient knowledge of nursing documentation and ENRS use contributed to incomplete records.	“… we still have insufficient knowledge …” (A2)
	Patient care and monitoring	Deviations from standards and limited supervision affected care quality.	“Staff do not always adhere to the established standards …” (B6)
Insufficient documentation of caring and holistic care	—	Documentation primarily focused on physiological needs, with limited recording of psychosocial and spiritual aspects.	“… social and psychological aspects only partially.” (A3)
Technological competency	Use of technology	Competent ENRS use involved retrieving, interpreting, and applying patient data to holistic care.	“… we incorporate the nursing process …” (B5)
	Education and training	Education and ongoing training supported the competent use of ENRS.	“… we received training …” (B5)
Caring and documentation	Caring behaviour	Caring was understood as addressing physiological, psychological, social, and spiritual needs.	“… caring behaviour for me means …” (A1)
	Education	Caring behaviour could be strengthened through education and team reflection.	“… caring behaviour could also be developed …” (A9)
	Standardisation of documentation	Standardised nursing language was essential for holistic and comparable documentation.	“… it is necessary to implement a standardised approach …” (B6)
Consequences	Individual level	Effects on patient safety, outcomes, and nurse workload.	“… reflected in the patient’s health status …” (A10)
	Organisational level	Impact on care quality, complaints, and legal consequences.	“… the hospital then must cover legal costs …” (A10)
	National level	Implications for continuity of care and public trust.	“… errors in care would occur less frequently …” (A10)

Legend: ENRS—Electronic Nursing Record System.

## Data Availability

For ethical and privacy considerations, and for the purposes of remaining in accordance with the approval provided by the institutional ethics committee, these data cannot be publicly shared. The restriction is necessary because the raw qualitative data contain sensitive and potentially identifiable information about the study participants.

## Abbreviations

The following abbreviations are used in this manuscript:

ENRS	Electronic Nursing Record System
EHR	Electronic Health Record
TCCN	Technological Competency as Caring in Nursing
MAXQDA	MAXimum Qualitative Data Analysis
COREQ	Consolidated Criteria for Reporting Qualitative Research

## Appendix A

**Table A1:** COREQ (Consolidated criteria for Reporting Qualitative research) Checklist [25].

Topic	Item No.	Guide Questions/Description	Reported on Page No.
Domain 1: Research team and reflexivity			
Personal characteristics			
Interviewer/facilitator	1	Which author/s conducted the interview or focus group?	p. 3
Credentials	2	What were the researcher’s credentials? E.g. PhD, MD	p. 3
Occupation	3	What was their occupation at the time of the study?	p. 3
Gender	4	Was the researcher male or female?	p. 1 and 3
Experience and training	5	What experience or training did the researcher have?	p. 3
Relationship with participants			
Relationship established	6	Was a relationship established prior to study commencement?	p. 3
Participant knowledge of the interviewer	7	What did the participants know about the researcher? e.g., personal goals, reasons for doing the research	p. 3
Interviewer characteristics	8	What characteristics were reported about the interviewer/facilitator? e.g., Bias, assumptions, reasons and interests in the research topic	p. 3
Domain 2: Study design			
Theoretical framework			
Methodological Orientation and Theory	9	What methodological orientation was stated to underpin the study? e.g., grounded theory, discourse analysis, ethnography, phenomenology, content analysis	p. 2
Participant selection			
Sampling	10	How were participants selected? e.g., purposive, convenience, consecutive, snowball	p. 3
Method of approach	11	How were participants approached? e.g., face-to-face, telephone, email	p. 3
Sample size	12	How many participants were in the study?	p. 5
Non-participation	13	How many people refused to participate or dropped out? Reasons?	p. 3
Setting			
Setting of data collection	14	Where was the data collected? e.g., home, clinic, workplace	p. 3
Presence of non- participants	15	Was anyone else present besides the participants and researchers?	p. 3
Description of sample	16	What are the important characteristics of the sample? e.g., demographic data, date	p. 5
Data collection			
Interview guide	17	Were questions, prompts, and guides provided by the authors? Was it pilot-tested?	pp. 3–4
Repeat interviews	18	Were repeat interviews carried out? If yes, how many?	p. 4
Audio/visual recording	19	Did the research use audio or visual recording to collect the data?	pp. 3–4
Field notes	20	Were field notes made during and/or after the interview or focus group?	pp. 3–4
Duration	21	What was the duration of the interviews or focus group?	p. 4
Data saturation	22	Was data saturation discussed?	p. 5
Transcripts returned	23	Were transcripts returned to participants for comment and/or correction?	pp. 4–5
Domain 3: analysis and findings			
Data analysis			
Number of data coders	24	How many data coders coded the data?	p. 4
Description of the coding tree	25	Did the authors provide a description of the coding tree?	p. 4
Derivation of themes	26	Were themes identified in advance or derived from the data?	p. 4
Software	27	What software, if applicable, was used to manage the data?	p. 4
Participant checking	28	Did participants provide feedback on the findings?	p. 4
Reporting			
Quotations presented	29	Were participant quotations presented to illustrate the themes/findings? Was each quotation identified? e.g., participant number	p. 5–15 (Section 3)
Data and findings consistent	30	Was there consistency between the data presented and the findings?	p. 5–15
Clarity of major themes	31	Were major themes clearly presented in the findings?	p. 5–15
Clarity of minor themes	32	Is there a description of diverse cases or a discussion of minor themes?	p. 5–15
